# Antibiotic appropriateness and adherence to local guidelines in perioperative prophylaxis: results from an antimicrobial stewardship intervention

**DOI:** 10.1186/s13756-020-00814-6

**Published:** 2020-10-26

**Authors:** Francesco Vladimiro Segala, Rita Murri, Eleonora Taddei, Francesca Giovannenze, Pierluigi Del Vecchio, Emanuela Birocchi, Francesco Taccari, Roberto Cauda, Massimo Fantoni

**Affiliations:** 1 Section of Infectious Diseases, Department of Safety and Bioethics, Catholic University of the Sacred Heart, Rome, Italy; 2 Dipartimento di Scienze di Laboratorio e Infettivologiche, Fondazione Policlinico Universitario Agostino Gemelli IRCCS, Rome, Italy

**Keywords:** Surgical prophylaxis, Antibiotic resistance, Antibiotic stewardship, Surgical-site infection

## Abstract

**Objectives:**

Surgical antibiotic prophylaxis (SAP) represents a major indication of antibiotic consumption worldwide. The present study aims to report the results of an enabling, long-term AMS intervention conducted between 2013 and 2019 on an Italian University Hospital performing more than 40.000 surgical interventions per year.

**Methods:**

SAP inappropriateness was defined according to the ASHP guidelines and divided in four main categories: *indication*, *selection and dosing, duration*, *timing*. Between 2013 and 2019, we conducted a continuative AMS intervention over 14 surgical departments that included enablement, review of selected clinical records and feedback.

**Results:**

We collected a total of 789 SAP prescribed to 735 patients (mean age 56.7 ± 17.8y). Overall, guideline adherence improved from 36.6% (*n* = 149) at baseline to 57.9% (*n* = 221) post-intervention (*P* <  0.0001). A significant improvement (*P* <  0.001) was also detected for each category: *indication* (from 58.5 to 93.2%), *selection and dosing* (from 58.5 to 80.6%), *timing* (from 92.4 to 97.6%), *duration* (from 71 to 80.1%).

**Conclusions:**

Though results cannot be generalized to all hospital populations, enabling AMS interventions may be effective in establishing a sustained improvement in SAP appropriateness rates. Once identified the main causes of SAP inappropriateness, tailored AMS interventions for each department may be beneficial. Further studies are needed to evaluate specific outcomes as incidence of surgical site infections and antimicrobial resistance.

## Background

Antimicrobial resistance is considered a global threat for both human health and economic development [1]. In both low-income and high-income countries, resistant microorganisms are the main causative agents of healthcare-associated infections (HAIs) and, among those, surgical site infections (SSIs) are the most reported and surveyed type of HAI, the accounting for up to 20% of all hospital acquired infections [2].. SSIs are defined as postoperative infections occurring within 30 days from a surgical procedure, or within 1 year from a permanent implant [3].

The European Centre for Disease Prevention and Control (ECDC) reports an in-hospital incidence ranging from 0.1 to 5.7 surgical site infections per 1000 post-operative patient-days. Cumulative incidence depends greatly on the type of surgical procedure, with highest rates described for open and laparoscopic colorectal operations (respectively 10.1 and 6.4%), followed by open cholecystectomy (3.9%) and coronary artery bypass graft (2.6%) [3]. Once occurred, surgical site infections are associated with a 2- to 11- fold increased risk of mortality, and the average length of stay is extended of 7–11 days [1, 4, 5]. Excess in healthcare cost is driven by prolonged hospitalization, treatment, additional diagnostic tests and re-operations [6]. The prevention of these infections is complex and it requires the integration of a range of measures before, during and after surgery [7].

Antimicrobial stewardship (AMS) programs play an increasingly important role in patient care and hospital policies [8]. They are widely recognized as a key intervention in reducing the burden of healthcare associated infections and costs, by both improving antimicrobial prescriptive habits and limiting the spread of resistance. Of note, 2016 guidelines by the World Health Organization highlighted the role of unnecessary prolongation of surgical antibiotic prophylaxis (SAP) in developing of antimicrobial resistance [7], and the implementation of AMS plan has recently been included among the Joint Commission Standards of Care [9].

The present study aims to report the results of an educational, participative, long-term continuing AMS intervention conducted between 2013 and 2019 on an Italian University Hospital performing more than 40.000 surgical interventions per year.

## Methods

### Clinical setting

The study was conducted on a 1.500-bed teaching hospital in Rome, Italy. SAP appropriateness survey and intervention implementation was performed within 14 surgical departments: digestive surgery, endocrine surgery, general surgery, hepato–pancreato–biliary surgery, urologic surgery, cardiac surgery, gynecologic surgery, neurosurgery, ear–nose–throat (ENT) surgery, orthopedic and spine surgery.

### Data collection and eligibility

Data about SAP were collected on two separate surveys, one at baseline (April 2013), and one after the long-term AMS intervention (*post-intervention,* April 2019). All adult patients (aged 18 or older) undergoing elective surgery were eligible for the study. Patients who underwent more than one intervention were considered as separate cases. All cases with suspected or confirmed infection prior to surgery were excluded, as well as outpatients and day-stay patients. Assessments were conducted by trained local surveyors (infectious diseases residents and interns) using a pre-defined collection data form. If no antibiotic prescription was recorded, it was assumed that antibiotics were not given.

The dataset included the following: baseline demographics (age, gender, admitting specialty, internal identification number, documented antimicrobial allergies), type of surgical procedure, antimicrobial usage (prescribed molecule, timing, dosage, route of administration), whether prophylaxis was prolonged for more than 24 or 48 h and, in the procedures lasting longer than 4 h, whether an adjunctive dose of antibiotic was administered or not (*re-dosing*).

### Definitions

For the purpose of the study, SAP appropriateness was evaluated according to ASHP, WHO and local guidelines [7, 10, 11]. Primary outcome of the study was the overall compliance to local guidelines. Inappropriateness was described according to the following dimensions, that were not considered mutually exclusive:

### Indication

Antibiotic administration for procedures in which SAP is not recommended (excess of indication) or lack of administration in cases where SAP is recommended (defect of indication);

### Selection and dosing

Prescription of an incorrect antimicrobial agent (spectrum too narrow or too broad) for the specific surgical procedure and for selected patient characteristics (i.e.: beta-lactam allergy). In the procedures lasting longer than 4 h, we underlined if short-acting antibiotics, like cefazolin, were administered in repeated doses.

### Timing

Administration of surgical prophylaxis outside the recommended time frame, that is, prior to 120 min before the incision, or anytime after the incision.

### Duration

Surgical prophylaxis prolonged for more than 24 h after surgery was considered inappropriate. In case of open-heart surgery, we considered inappropriate if prophylaxis was prolonged for more than 48 h, or discontinued before than 24 h from the incision [12].

### Intervention

Between 2013 and 2019, we conducted a continuative, long-term, enabling AMS intervention within all the surgical departments included in the study (see above). The intervention consisted in a series of structured audit meetings, lasting at least 1 h, including all figures involved in SAP prescription (i.e.: surgeons and anesthesiologists). In April 2014, 4 months after the first survey period, appropriateness results were discussed with prescribing personnel of each department, thus giving a detailed feedback of SAP prescriptive performance.

All along the study period, selected clinical records review and evaluation was conducted according to the NHS Institute for Clinical Excellence (NICE) methodology [13]. In this setting, AMS team implemented a jointed review of international guidelines, thus encouraging prescribers to actively participate to the revision of local SAP recommendations. AMS team involved in the audit meeting included, along with an Infectious Diseases specialist, a surgeon, a pharmacist and a member of the Clinical Governance staff.

So far, no restrictive intervention has been implemented.

### Statistical analysis

Categorical variables and proportions were summarized and compared between groups using a *x*^2^ test. Means and standard deviations were used to describe continuous variables. A two-tailed Student’s t test was performed to compare continuous variables. A *P*-value of < 0.05 (two-tailored) was deemed to be statistically significant. Statistical analyses were performed using Statistical Package for the Social Sciences Version 26.0 software (IBM Corp., Armonk, NY).

## Results

We analyzed a total of 789 SAP prescriptions administered to 735 patients, respectively 407 and 382 in pre- and post-interventional surveys (2013 and 2019). Demographic factors and admitting surgical departments are summarized in Table 1. Mean age was 56.7 year (± 17.8y), and 58.3% (*n* = 433) of included patients were female. 13.3% (*n* = 104) of all surveyed cases involved contaminated or dirty operative wounds

**Table 1:** Demographic variables and patients characteristics before and after intervention

Variable	Total sample	Baseline	Post-intervention
Cases, n	789 (100)	407 (51.6)	382 (48.4)
Age, mean (SD) year	56.7 (17.8)	52.5 (18.5)	61.2 (16)
Females, n (%)	465 (58.9)	237 (58.3)	229 (60.1)
Reported allergy to beta-lactams, n (%)	15 (1.9)	6 (1.4)	9 (2.3)
Surgical procedure, n (%)			
Cardiac surgery	61 (7.7)	14 (3.4)	47 (12.3)
Digestive surgery	95 (12.0)	35 (8.6)	60 (15.7)
Endocrine surgery	78 (9.9)	61 (15.0)	17 (4.5)
Vertebral surgery	8 (1)	5 (1.2)	3 (8)
Hepato–pancreato–biliary surgery	19 (2.4)	15 (3.7)	4 (1)
Gynecologic surgery	128 (16.2)	74 (18.2)	54 (14.1)
Neurosurgery	81 (10.3)	28 (6.9)	53 (13.9)
Ear–nose–throat surgery	104 (13.2)	57 (14.0)	47 (12.3)
Orthopedic surgery	116 (14.7)	72 (17.7)	44 (11.5)
Urology	99 (12.5)	46 (11.3)	53 (13.9)
Surgical wound class, n (%)			
Clean	488 (61.9)	242 (59.5)	246 (64.4)
Clean-contaminated	197 (25)	140 (34.4)	57 (14.9)
Contaminated	85 (10.8)	21 (5.2)	64 (16.8)
Dirty	19 (2.5)	4 (1)	15 (3.9)

In 2013, inappropriateness was almost entirely multi-dimensional (Table 2): 98.8% of inappropriate antimicrobial prescriptions were non adherent to local guidelines for at least two dimensions. Before the intervention, the most common reason of inappropriateness were mistakes in *indication:* 169 (41.5%) of surveyed cases were either prescribed when unnecessary or were not administered prior to procedures with defined SAP indication. In addition, the same amount of patients didn’t receive the correct antimicrobial agent for SAP, or they an inappropriate dose. Furthermore, 118 (29%) of the surveyed baseline cases were inappropriately prolonged after the completion of the surgical procedure (for more than 48 h in case of open-heart surgery, or more than 24 h for all other surgeries). Of note, in 2013, in all surveyed cases of prolonged SAP prescription there was at least one additional reason of inappropriateness.

**Table 2:** SAP appropriateness

Variable	Total sample	2013	2019	*p value*
Overall appropriateness^a^, n (%)	370 (46,9)	149 (36,6)	221 (57,9)	< 0,0001
Appropriateness in *indication*^b^	594 (75,3)	238 (58,5)	356 (93,2)	< 0,0001
Appropriateness in *selection and dosing*^c^	546 (69,2)	238 (58,5)	308 (80,6)	< 0,0001
Appropriateness in *timing*^d^	749 (94,9)	376 (92,4)	373 (97,6)	= 0,001
Appropriateness in *duration*^e^	595 (75,4)	289 (71)	306 (80,1)	= 0,002
Multi-dimensional inappropriateness^f^				
within total cases, %	28,1 (221/789)	44,7 (182/407)	10,2 (39/382)	< 0,0001
within inappropriate cases, %	52,7 (221/419)	70,5 (182/258)	24,2 (39/161)	

^a^ SAP prescriptions fully adherent to local guidelines

^b^ SAP administered only if indicated in local guidelines or not administered if not recommended for the specific surgical

procedure

^c^ Correct choice and dosing of the antimicrobial agent, including repeated doses in cases of procedures lasting > 4 h

^d^ SAP administered within 120 min from the incision

^e^ SAP discontinued within 24 h from surgery, or within 48 h from open-heart surgery

^f^ SAP prescriptions non adherent to local guidelines for ≥2 dimensions

**Fig. 1:**
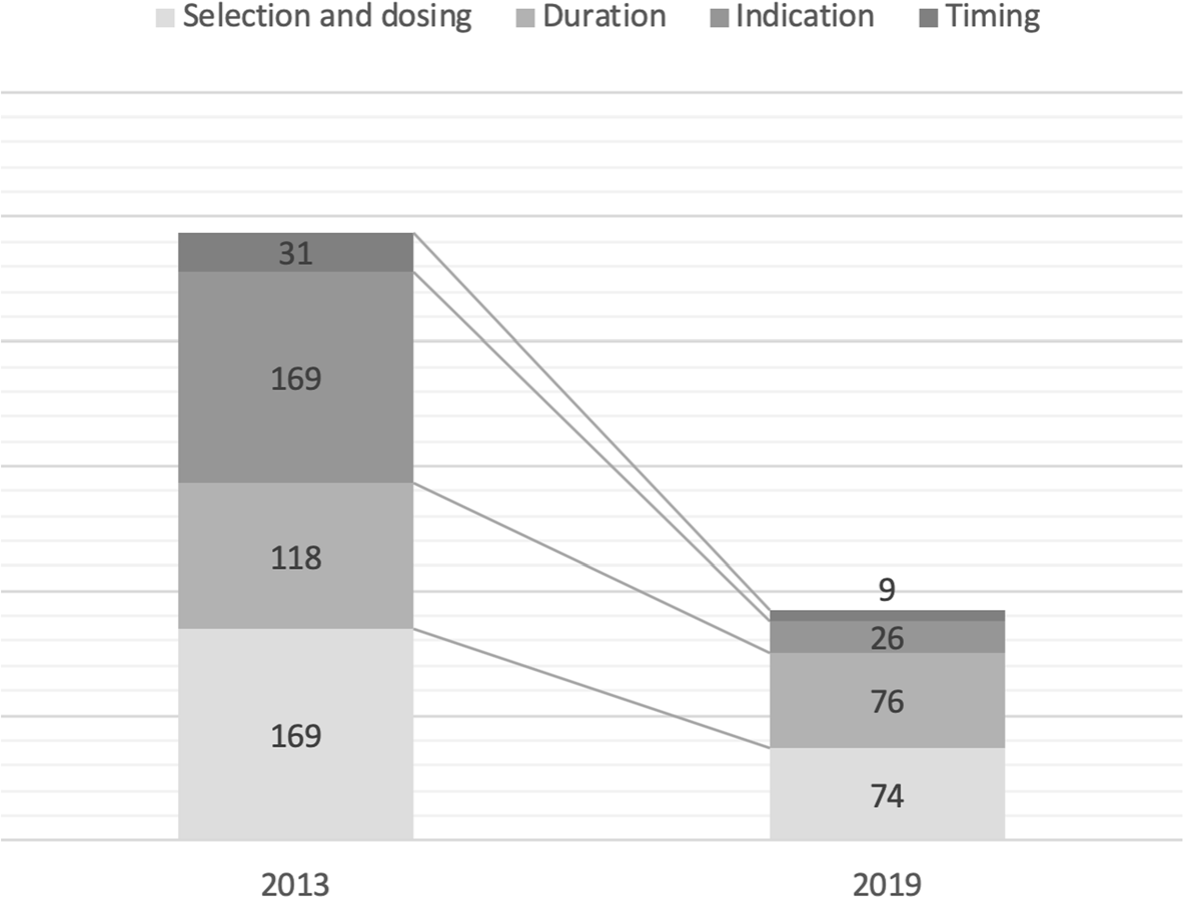
Total number of inappropriate cases per dimension at baseline and post-interventional survey

In the post-interventional survey, appropriateness in *duration* shifted from 71 to 80.1% (*P* <  0.0001) of SAP prescriptions, while appropriateness in *indication* passed from 58.5 to 93.2% (P <  0.0001). A significant increase (*P* <  0.05) in prescriptive appropriateness was detected also for the other categories included in the study: the correct antimicrobial agent was prescribed in 80.6% of post-intervention cases versus 58.5% of the baseline cases, and surgical prophylaxis was administered at the recommended timing in 97.6% vs 92.4% of cases. Overall, guideline adherence was 46.9% (*n* = 370), improving from 36.6% (*n* = 149) at baseline to 57.9% (*n* = 221) post-intervention (*P* <  0.0001). Overall and dimension-specific drop in inappropriateness is showed in Fig. 1.

## Discussion

Hospital-associated infections and antimicrobial resistance are a public health priority, and there is a considerable amount of evidence assessing the efficacy of AMS interventions in decreasing the burden of antimicrobial resistance [14]. According to the existing body of evidence, both restrictive and enabling interventions demonstrated to be effective in increasing compliance with antibiotic policies without an increase in mortality, hospital length-of-stay or rate of drug-related adverse events [15]. Still, suboptimal use of antimicrobial for surgical prophylaxis is a common phenomenon [16].

In our study, overall inappropriateness dropped from 63.4 to 42.1%, thus lowering post-interventional results to values reported in countries with low-rates of antimicrobial resistance [17, 18]. However, it should be noted that few studies addressed multiple outcomes of SAP guidelines adherence (*indication, antimicrobial selection and dosing, timing, duration*) targeting several surgical specialties. Furthermore, in our work, the total guideline adherence rates are affected form the fact that we defined as “overall appropriate” only prescriptions in which all the dimensions were respected.

At baseline, inappropriateness was largely multidimensional: in 70.5% of pre-interventional non-adherent prescriptions, at least two reasons of inappropriateness were recorded, while this percentage fell to 24.2% in 2019.

In the latter, it persisted almost exclusively for urologic and ENT surgical procedures, departments that were identified as main drivers of post-interventional overall inaccuracy. This leads to two considerations. First, that without AMS team guidance, SAP prescriptions tend to be more chaotic, being *selection and dosing* and *duration* the two most commonly associated types of inappropriateness (wrong choice/dosing of the antimicrobial agent plus prolonged administration); in contrast, after enablement, only a small proportion of non-compliant departments fail to follow system processes and guidelines. Second, that the same enabling intervention didn’t affect all departments with the same strength. This may be due to inner difficulties in shifting from old, unsupported practices to new prescriptive behaviors, or it may be due to a wider, department-specific deregulation of SAP prescribing. However, a detailed investigation of the reasons behind lack of compliance fall outside the aim of this work.

As recently stated from a Cochrane Library editorial [15], the effectiveness of antimicrobial stewardship needs to be assessed in reason of long-term outcomes, in order to differentiate the impact of a specific intervention with the unintended effects of other concurrent factors. Furthermore, evaluation of sustainability is particularly attractive, because unmaintained results prevent AMS resources to focus towards new priorities. Restriction is relatively low cost, but evidence exist that removal of restrictive measures could lead to reversal of intervention effects [19, 20], probably in reason of a loss of trust and guidance between SAP prescribers and AMS team. More concerning, however, are the findings that enablement fail to achieve a sustained effect after implementation [21]. In our study, 6 years after the baseline evaluation, SAP prescribing still showed a substantial improvement (Fig. 1), highlighting the effectiveness of enablement (review and recommend change) also in terms of sustainability.

The present study evaluates the impact of a continuous, enabling intervention 6 years after its implementation, focusing exclusively on surgical prophylaxis guideline compliance. In our analysis we didn’t include secondary outcomes (e.g. mortality, length of stay) or other clinical (e.g. surgical site infection or acute kidney injury) and microbial outcomes (CDI, colonization or infection with antimicrobial-resistant bacteria), which could have represented an unintended consequence of a generalized improvement in clinical care, rather than a direct measure of the effectiveness of our intervention.

Our study has several limitations. First, our intervention didn’t include explicit goal setting and action planning. Second, we didn’t include a control group, as this was considered unethical. Third, the lack of secondary clinical outcomes and of microbial data didn’t allow us to determine the actual impact of the SAP prescriptive improvement.

Finally, in 2019, appropriateness in *duration* was still far to be optimal, being the most reported reason of non-compliant prescribing in the post-interventional survey, as well as the dimension with the lowest relative improvement rate (from 71% in 2013 to 80,1% in 2019; *P* = 0.002). However, this is not an exclusive finding of this work. While all current guidelines recommend SAP discontinuation within 24 h from incision, globally, surgeons still have a tendency to routinely continue SAP several days after surgery [22, 23]. This behavior is likely sustained by the belief that prolonged antimicrobial administration is safe and more efficient in reducing SSI incidence [24, 25], this despite evidence of SAP ineffectiveness beyond 24 h [7, 11]. Conversely, as highlighted by the World Health Organization, prolongation of antibiotic prophylaxis is one of the major determinants of antimicrobial resistance [7, 26].

## Conclusions

Antibiotic resistance is a major public health concern. Antibiotic stewardship programs are changing the entire healthcare landscape, having largely demonstrated to be effective and safe. In surgery, proper administration of antibiotic prophylaxis is crucial in preventing the incidence of SSI, but it requires constant effort and close collaboration among SAP prescribers and AMS team. Due to its toxicity and to its probable impact on local ecology, future AMS intervention will need to prioritize SAP prolongation improvement rates.

## Data Availability

The datasets used and/or analyzed during the current study are available from the corresponding author on reasonable request.

## Abbreviations

HAI: Hospital-acquired infection
SSI: Surgical site infection
AMS: anti-microbial stewardship
ENT: ear-nose-throat
ECDC: European Centre for Disease Prevention and Control
WHO: World Health Organization
ASHP: American Society of Health-System Pharmacists
NHS: United Kingdom National Health Service
